# Integrated analysis of gene expression and DNA methylation profiles in ovarian cancer

**DOI:** 10.1186/s13048-020-00632-9

**Published:** 2020-03-19

**Authors:** Guanghui Gong, Ting Lin, Yishu Yuan

**Affiliations:** 1 Department of Pathology, Xiangya Hospital, Central South University, 87 Xiangya Road, Changsha, Hunan 410008 People’s Republic of China; 2 Institute of Reproductive and Stem Cell Engineering, School of Basic Medical Science, Central South University, Changsha, 410078 People’s Republic of China; 3 Hunan Provincial Key Laboratory for the Prevention and Treatment of Ophthalmology and Otolaryngology Diseases with Traditional Chinese Medicine, Changsha, 410208 People’s Republic of China; 4 Hunan Provincial Key Discipline of Chinese Head and Neck Science, Changsha, 410208 People’s Republic of China

**Keywords:** Ovarian cancer, Methylation, Gene expression

## Abstract

**Background:**

Ovarian cancer is an epithelial malignancy that intrigues people for its poor outcome and lack of efficient treatment, while methylation is an important mechanism that have been recognized in many malignancies. In this study, we attempt to assess abnormally methylated gene markers and pathways in ovarian cancer by integrating three microarray datasets.

**Methods:**

Three datasets including expression (GSE26712 and GSE66957) and methylation (GSE81224) datasets were accessed. GEO2R platform was used to detect abnormally methylated-differentially expressed genes. Protein-protein interaction (PPI) networks were built and analysed for hypermethylated and hypermethylated differentially expressed genes using Cytoscape software and Mcode app. GEPIA and cBioPortal platforms were used to validate the expression of the hub genes and the correlation between their mRNA expressions and methylation levels. Kaplan Meier-plotter platform were used to assess the prognostic significance of the hub genes.

**Results:**

Six hundred eighty-one hypomethylated-upregulated genes were detected and involved in Rap1 signaling pathway, biosynthesis of amino acids, endocrine resistance, apoptosis, pathways in cancer. The hub genes were TNF, UBC, SRC, ESR1, CDK1, PECAM1, CXCR4, MUC1, IKBKG. Additionally, 337 hypermethylated-downregulated genes were detected and involved in pathways in cancer, focal adhesion, sphingolipid signaling pathway, EGFR tyrosine kinase inhibitor resistance, cellular senescence. The hub genes were BDNF, CDC42, CD44, PPP2R5C, PTEN, UBB, BMP2, FOXO1, KLHL2. TNF, ESR1, MUC1, CD44, PPP2R5C, PTEN, UBB and FOXO1 showed significant negative correlation between their mRNA expressions and methylation levels. TNF, ESR1 and FOXO1 showed prognostic significance.

**Conclusions:**

Two novel gene networks were found for ovarian cancer. TNF, ESR1, MUC1 and FOXO1 are our candidate genes that might take part in ovarian cancer progression in an epigenetic approach, TNF, ESR1 and FOXO1 may serve as potential markers for ovarian cancer prognosis evaluation.

## Background

Ovarian cancer, an epithelial malignancy, is reported to be the most common lethal malignancy among gynecologic cancer [1]. And the outcome of the patients is poor due to late diagnosis for lack of early signs and symptoms [2]. Ovarian cancer is prone to metastasis and recurrence [3], but the pathogenesis is still unclear, the suspected etiology include ovulation, hormones, genetics, environmental factors [4–6].

The standard treatment for ovarian cancer include surgery and chemotherapy, other treatments include radiation, hormone, immunotherapy, but the survival rate for it is still low (https://www.cancerresearchuk.org/about-cancer/ovarian-cancer/survival) due to advanced stage when diagnosed and frequent recurrence (which often accompanied with increases chemoresistance) [5], thus more attention should be paid to ovarian cancer.

DNA methylation, the most studied epigenetic mechanism, is reported to be related to mRNA and miRNA expression regulation, thus contribute to cancer initiation or progression [7]. Recently, more and more studies indicated that abnormal gene methylation in promoter regions is involved with chemical therapy and targeting therapy of ovarian cancer [8–10].

In this study, DNA methylation datasets in ovarian cancer were screened. A series of bioinformatics tools were used for integrated analysis and detection of hub genes. Then the levels of hub genes, and the correlation between their expression and methylation level of them were confirmed by GEPIA [11], and cBioPortal [12, 13] platforms, with the data from The Cancer Genome Atlas (TCGA) and GTEx (The Genotype-Tissue Expression). These confirmed genes are our candidate genes for a deeper study later on in ovarian cancer progress.

## Methods

### Gene datasets and differentially expressed genes identification

Gene expression datasets: GSE26712 (185 ovarian cancer samples, 10 normal samples), GSE66957 (57 ovarian cancer samples, 12 normal samples) and gene methylation datasets: GSE81224 (10 ovarian cancer samples, 5 normal samples) were screened from Gene Expression Omnibus (GEO) database (http://www.ncbi.nlm.nih.gov/geo/) for the biggest number of cases and the data provided are normalized that no further adjustment is needed. GEO2R platform were used to detect differentially expressed genes (DEGs) and abnormally methylated genes between normal and ovarian cancer samples. The parameter for DEGs and abnormally methylated genes were set with |t| > 2, *P* < 0.05. Then the DEGs and abnormally methylated genes were processed in Funrich software (http://www.funrich.org) for integrated analysis and Venn diagram visualization.

### PPI network construction and modular analysis

STRING was used to build the protein-protein interaction (PPI) network (https://string-db.org/) [14], with minimum required interaction score set in 0.4. The result data was imported into Cytoscape [15] for visualization, subsequently, app Mcode (i.e., Molecular Complex Detection) was used to perform module analysis (Node score cut off > 3.5) for the PPI network complex. Hub genes were determined by connectivity degree > 27 for the hypomethylated-upregulated (HOUP) genes and connectivity degree > 12 for the hypermethylated-downregulated (HEDW) genes, the calculation of connection degrees of genes was performed using Microsoft Office Excel.

### GO and Reactome, KEGG pathway enrichment analysis

The STRING website described above also helped in the integrated analysis of biological meaning of the proteins (or genes), Gene Ontology (GO) analysis (including the biological process (BP), cellular component (CC), and molecular function (MF)), Reactome pathway and the Kyoto Encyclopedia of Genes and Genomes (KEGG) pathway enrichment analysis were conducted for the selected genes, *P* value < 0.05 was considered statistically significant, and top 10 of BP, CC, MF items and top 5 of KEGG, Reactome pathways were illustrated using a web tool (http://www.ehbio.com/ImageGP/index.php/Home/Index/index.html).

### Validation of the expression of hub genes and correlations between methylation and mRNA levels in TCGA samples

GEPIA platform was used to validate the expression levels of the hub genes and |log2FC| > 1 and *P* value < 0.05 was considered statistically significant. Methylation data TCGA 27 k methylation data were selected to assess the correlations between methylation levels and corresponding mRNA levels using cBioPortal platform (TCGA 450 k methylation data were not chosen because it contains only10 cases).

### Prognostic analysis of hub genes in ovarian cancer tissue samples

The overall survival (OS), progression-free survival (PFS) and post progression survival (PPS) curves of each hub gene were drawn using the online platform, the Kaplan Meier-plotter (http://kmplot.com/analysis/index.php) [16]. Both logrank *P* value and Hazard Ratio (HR, and 95% confidence intervals) were evaluated, *P* < 0.05 was considered statistically significant.

## Results

### Detection of abnormal methylated-differentially expressed genes in ovarian cancer

**Fig. 1:**
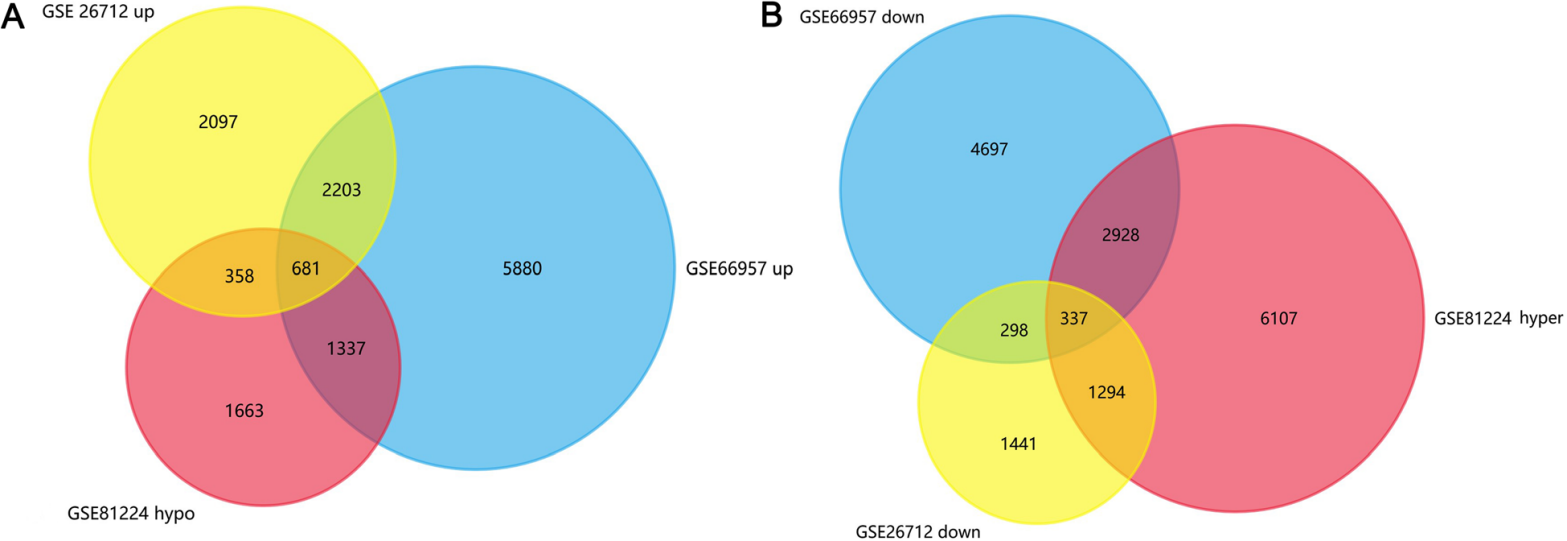
The integrated analysis of abnormal DNA methylation genes in ovarian cancer. The gene counting numbers were summarized in gene expression datasets (GSE26712, GSE66957) and gene methylation dataset (GSE81224), respectively. Left panel (**a**) was represented the hypomethylation and up-regulated genes, while right panel (**b**) represented the hypermethylation and down-regulated genes

GSE26712 and GSE66957 (expression datasets) and GSE81224 (methylation datasets) were analyzed with GEO2R, two Veen diagrams were drawn and 681 HOUP genes and 337 HEDW genes were detected from these three datasets (Fig. 1).

### Biological function and pathways enrichment analysis

The HOUP and HEDW genes were imported into STRING, respectively. Biological classification was evaluated for the two lists of genes including GO term enrichment analysis, KEGG pathways and Reactome pathways enrichment analysis.

**Fig. 2:**
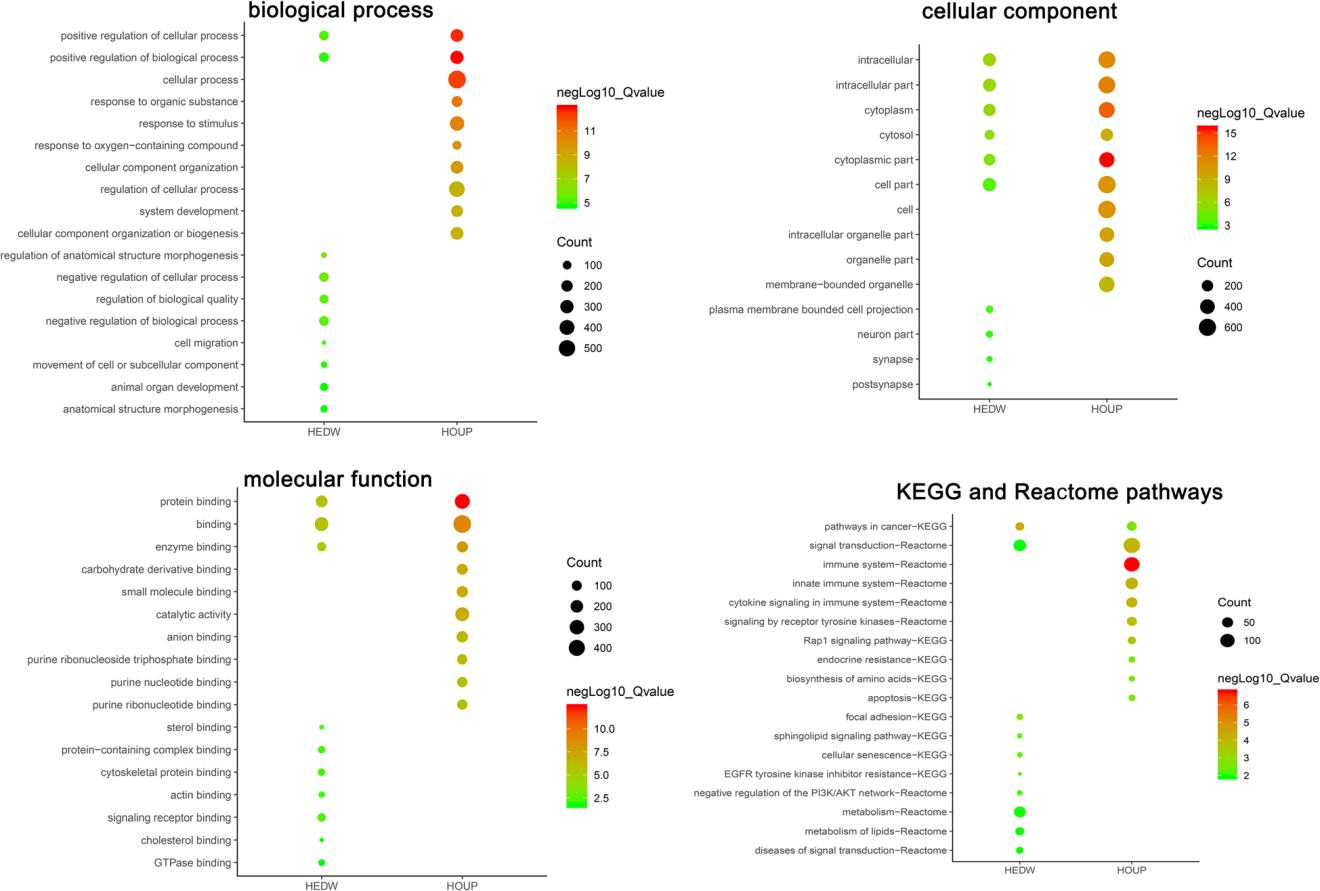
GO and KEGG pathway enrichment analysis. Biological process, cellular component, molecular function, KEGG and Reactome pathway analysis were presented. The size of each circle indicates the counting number on each part, while the color represents the *P*-value of the enrichment analysis

The HOUP genes were enriched in biological process such as positive regulation of biological process, positive regulation of cellular process, cellular process, response to organic substance, response to stimulus. GO cell component analysis showed that the genes were significantly enriched in cytoplasmic part, cytoplasm, intracellular part, intracellular, cell. GO molecular function analysis showed significantly enrichment in.

protein binding, binding, enzyme binding, carbohydrate derivative binding, catalytic activity. KEGG and Reactome pathways analysis indicated enrichment in Rap1 signaling pathway, biosynthesis of amino acids, endocrine resistance, apoptosis, pathways in cancer, immune system, innate immune system, cytokine signaling in immune system, signal transduction, signaling by receptor tyrosine kinases (Fig. 2).

The HEDW genes were enriched in biological process such as regulation of anatomical structure morphogenesis, negative regulation of cellular process, negative regulation of biological process, regulation of biological quality, positive regulation of cellular process. GO cell component analysis showed that the genes were significantly enriched in intracellular, cytoplasm, intracellular part, cytosol, cytoplasmic part. GO molecular function analysis showed significantly enrichment in binding, protein binding, enzyme binding, actin binding, cytoskeletal protein binding. KEGG and Reactome pathways analysis indicated enrichment in pathways in cancer, focal adhesion, sphingolipid signaling pathway, EGFR tyrosine kinase inhibitor resistance, cellular senescence, negative regulation of the PI3K/AKT network, metabolism, metabolism of lipids, signal transduction, diseases of signal transduction (Fig. 2).

### PPI networks and module analysis

**Fig. 3:**
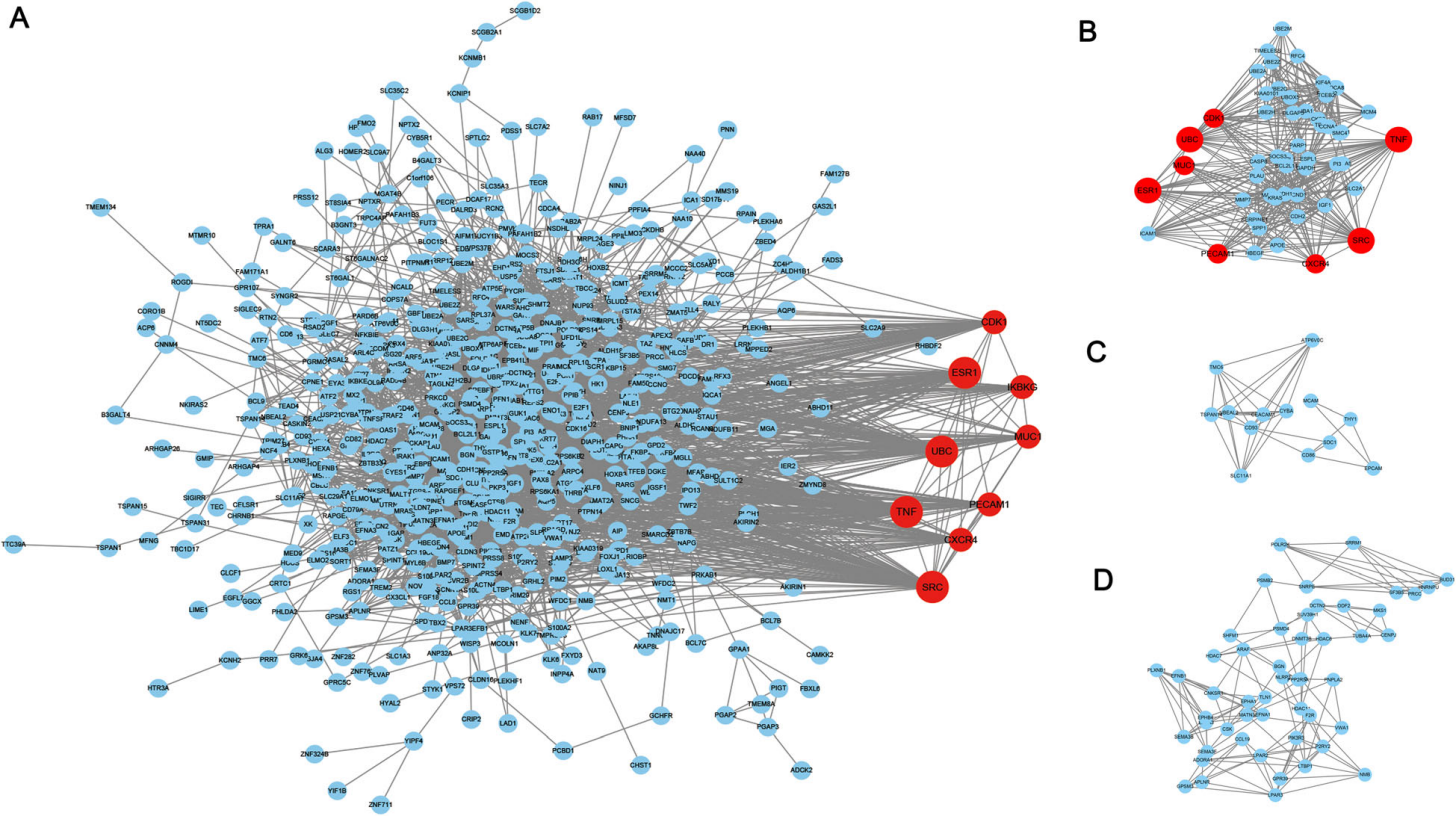
Protein-protein interaction (PPI) network and top three modules of the HOUP genes. **a** The PPI networks. The size of node indicated the connection degree value. Dark nodes represented hub genes. **b**-**d** Module 1 consists of 53 nodes, module 2 consists of 13 nodes and module 3 consists of 51 nodes

The PPI network graph for the HOUP genes was illustrated in Fig. 3a, 677 nodes and 3349 edges were included in the network. Nine nodes with the highest connectivity degrees were judged as hub genes, including tumor necrosis factor (TNF), polyubiquitin-C (UBC), proto-oncogene tyrosine-protein kinase (SRC), estrogen receptor 1 (ESR1), Cyclin-dependent kinase 1 (CDK1), platelet endothelial cell adhesion molecule (PECAM1), C-X-C chemokine receptor type 4 (CXCR4), mucin-1.

(MUC1), inhibitor of nuclear factor kappa B kinase regulatory subunit gamma (IKBKG). Three significant modules (Fig. 3b-d) were detected using app Mcode. Function analysis of the top three module genes using STRING showed enrichment in positive regulation of nitrogen compound metabolic process, positive regulation of macromolecule metabolic process, positive regulation of cellular metabolic process, positive regulation of cellular protein metabolic process, regulation of cellular process.

**Fig. 4:**
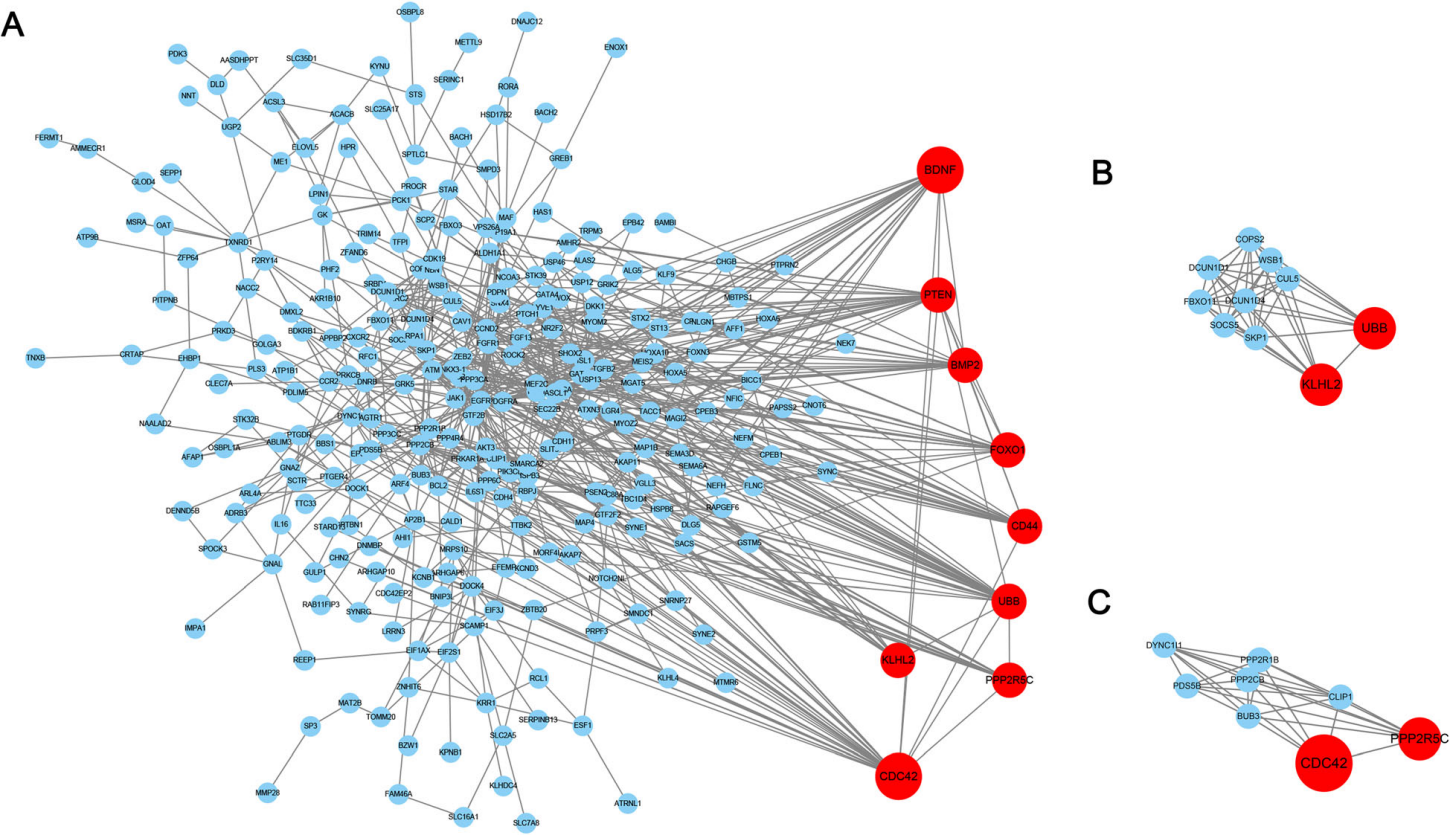
Protein-protein interaction (PPI) network and top three modules of the HEDW genes. **a** The PPI networks. The size of node indicated the connection degree value. Dark nodes represented hub genes. **b-c** Module 1 consists of 10 nodes and module 2 consists of 8 nodes

The PPI network graph for the HEDW genes was illustrated in Fig. 4a, 334 nodes and 772 edges were included in the network. Nine nodes with the highest connectivity degrees were judged as hub genes, including brain-derived neurotrophic factor (BDNF), cell division control protein 42 homolog (CDC42), CD44 antigen (CD44), Serine/threonine-protein phosphatase 2A 56 kDa regulatory subunit gamma isoform (PPP2R5C), phosphatase and Tensin homolog (PTEN), polyubiquitin-B (UBB), bone morphogenetic protein 2 (BMP2), forkhead box protein O1 (FOXO1), Kelch-like protein 2 (KLHL2). Two significant modules (Fig. 4b-c) were detected by app Mcode. Function analysis showed enrichment in protein modification by small protein conjugation, post-translational protein modification, protein ubiquitination, protein polyubiquitination, mitotic cell cycle.

### The validation of hub genes expression levels in TCGA ovarian cancer samples

**Fig. 5:**
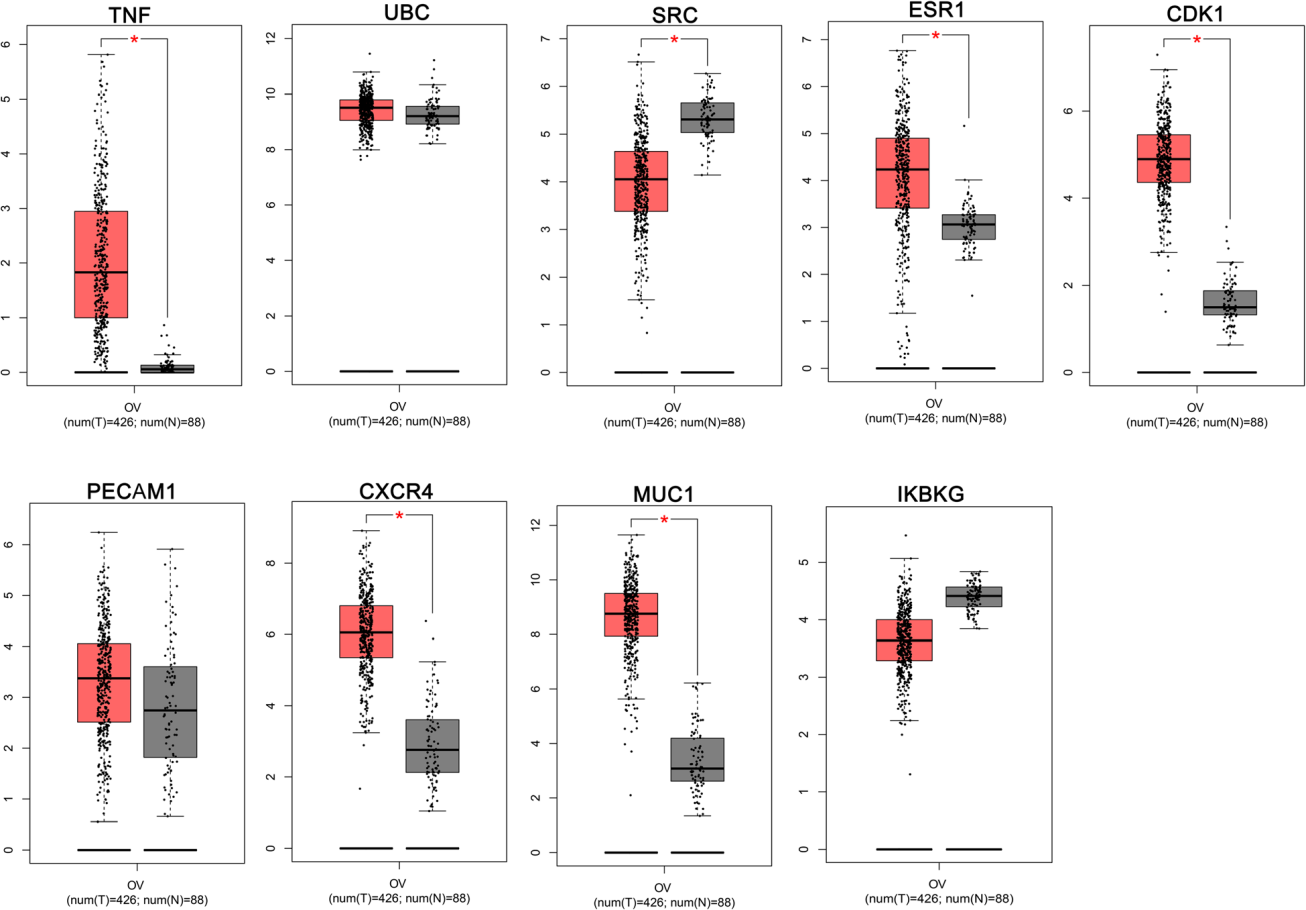
The mRNA expression levels of the HOUP hub genes (analyzed by GEPIA platform). TNF, ESR1, CDK1, CXCR4 and MUC1 were confirmed significantly higher in ovarian cancer samples than that in normal samples

**Fig. 6:**
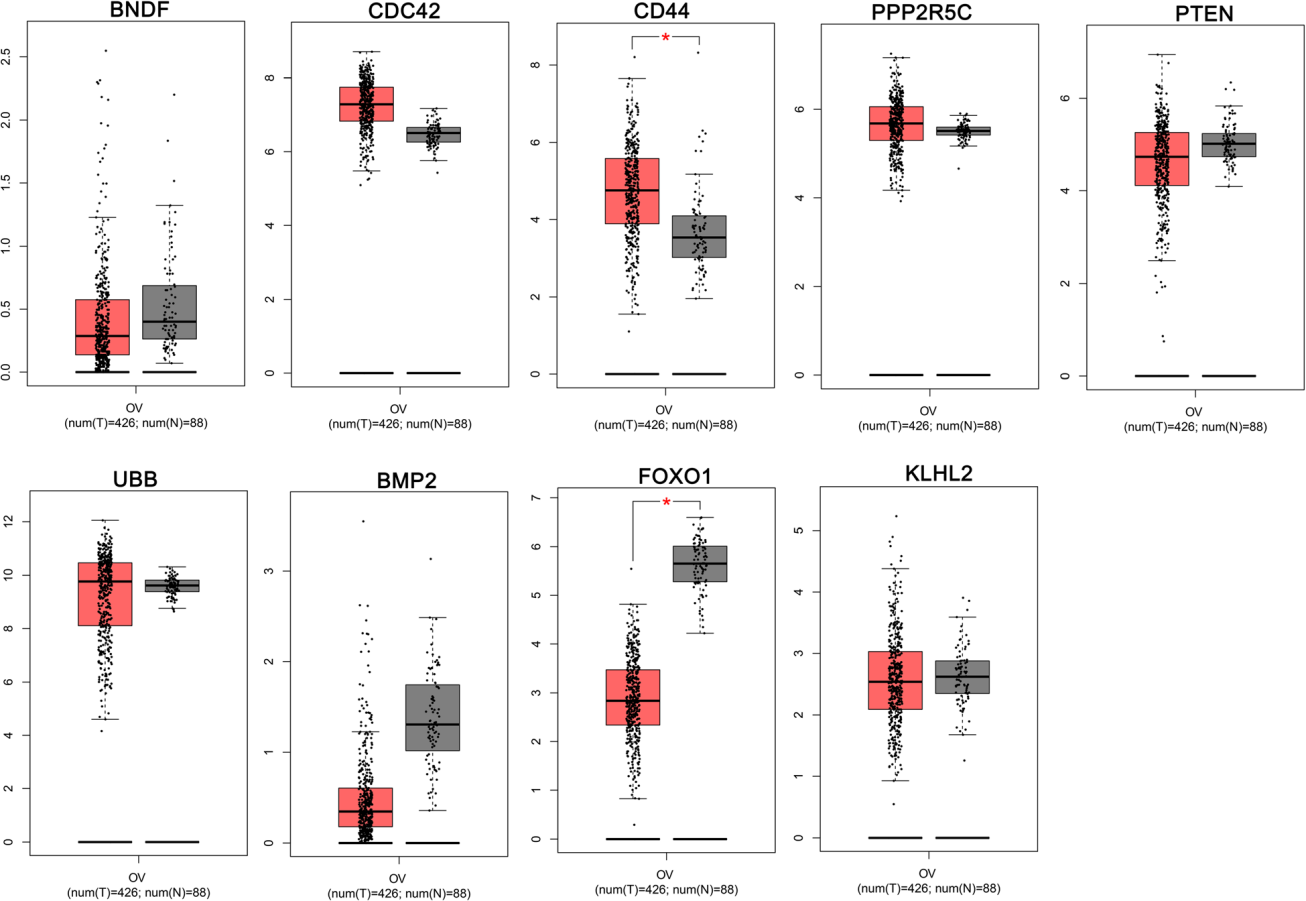
The mRNA expression levels of the HEDW hub genes (analyzed by GEPIA platform). FOXO1 was significantly lower in ovarian cancer tissues than that in normal tissues

To validate the expression levels of the HOUP and HEDW hub genes, GEPIA platform was used with the data origin from TCGA and GTEx. The results of the HOUP hub genes confirmed that the expression levels of TNF, ESR1, CDK1, CXCR4 and MUC1 (Fig. 5), indicating these 5 hypomethylated genes were activated in ovarian cancer development. For the HEDW hub genes, FOXO1 was significantly lower in ovarian cancer tissues than that in normal tissues (Fig. 6), indicating it might participate in ovarian cancer development with a different approach.

### Methylation level and mRNA expression level correlation analysis of the hub genes in ovarian cancer

For further validation of the effect of methylation on mRNA expression, we performed a correlation analysis for the two lists of hub genes with cBioPortal, negative correlations were found in TNF, ESR1, MUC1, CD44, PPP2R5C, PTEN, UBB and FOXO1 (Table 1) between DNA methylation levels and corresponding mRNA levels.

**Table 1:** Methylation analysis of hub genes in TCGA ovarian cancer. Negative correlations were found between TNF, ESR1, MUC1, CD44, PPP2R5C, PTEN, UBB and FOXO1 mRNA levels and methylation levels

Gene	correlations of methylation level and mRNA level (cBioportal)
TNF	Spearman: − 0.38(*p* = 2.16e-18)
	Pearson: − 0.38(*p* = 7.38e-18)
UBC	Spearman: 0.01(*p* = 0.799)
	Pearson: 0.04(*p* = 0.353)
SRC	No data
ESR1	Spearman: − 0.24(*p* = 1.13e-7)
	Pearson: −0.24(*p* = 1.04e-7)
CDK1	No data
PECAM1	No data
CXCR4	Spearman: −0.05(*p* = 0.263)
	Pearson: −0.05(*p* = 0.257)
MUC1	Spearman: − 0.13(*p* = 3.288e-3)
	Pearson: − 0.34(*p* = 9.48e-15)
IKBKG	No data
BDNF	Spearman: −0.04(*p* = 0.435)
	Pearson: 0.03(*p* = 0.516)
CDC42	Spearman: −0.09(*p* = 0.0469)
	Pearson: − 0.08(*p* = 0.0702)
CD44	Spearman: − 0.22(*p* = 5.88e-7)
	Pearson: − 0.22(*p* = 8.34e-7)
PPP2R5C	Spearman: − 0.20(*p* = 1.194e-5)
	Pearson: − 0.13(*p* = 4.291e-3)
PTEN	Spearman: − 0.18(*p* = 5.541e-5)
	Pearson: − 0.23(*p* = 3.32e-7)
UBB	Spearman: − 0.69(*p* = 6.66e-70)
	Pearson: − 0.81(*p* = 1.76e-116)
BMP2	Spearman: − 0.00(*p* = 0.944)
	Pearson: −0.11(*p* = 0.0150)
FOXO1	Spearman: − 0.16(*p* = 4.634e-4)
	Pearson: − 0.17(*p* = 1.385e-4)
KLHL2	No data

Taken together, TNF, ESR1, MUC1 and FOXO1 are selected as our candidate genes for further research.

### The prognostic significance of validated hub genes in ovarian cancer

**Fig. 7:**
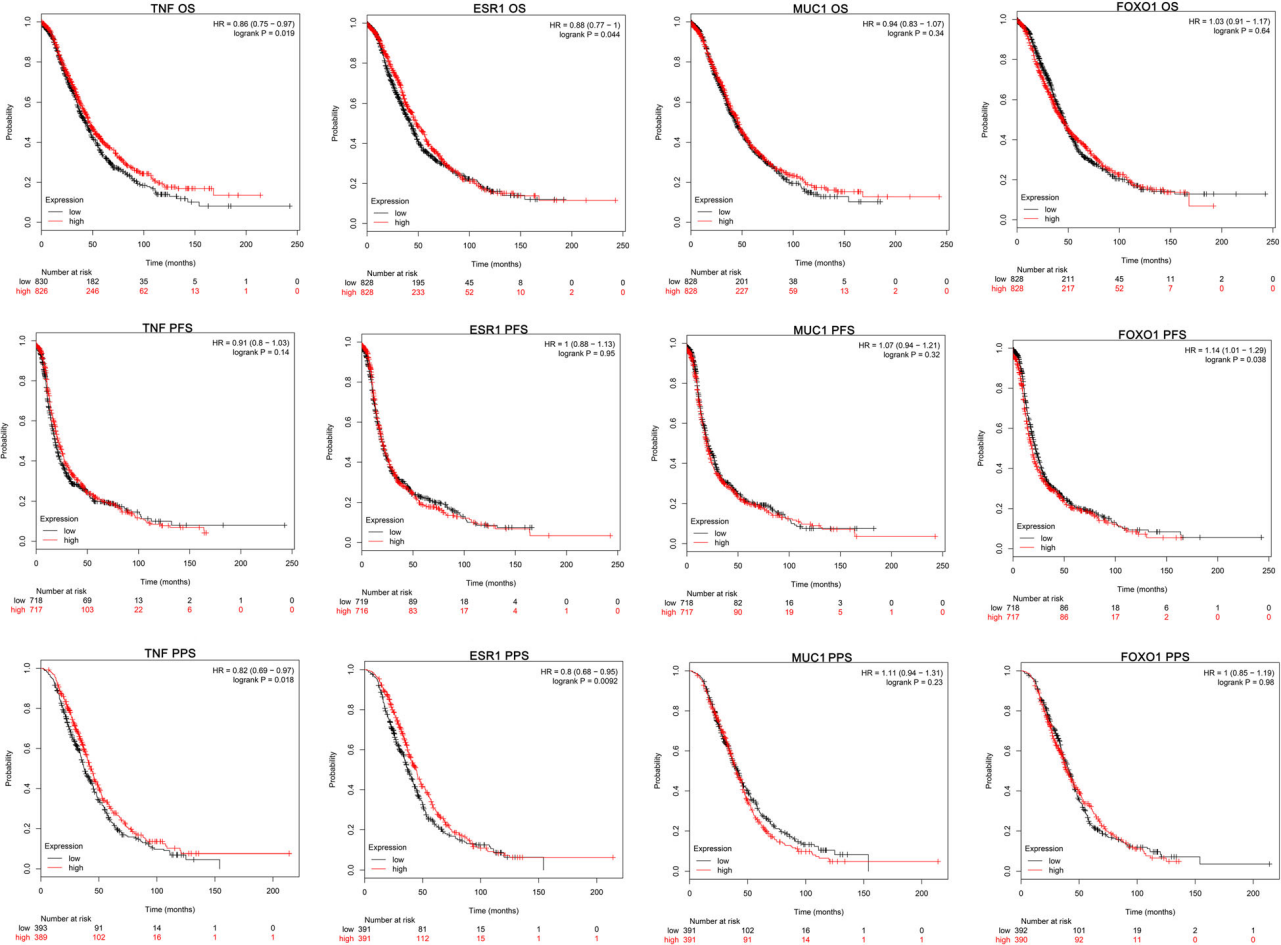
The prognostic significance of validated hub genes in ovarian cancer. The analysis results showed that higher level of TNF is related to longer OS and PPS time, and higher level of ESR1 and lower level of FOXO1 are potential protective factors for ovarian cancer patients’ survival (judged by both *P* value and 95% confidence intervals of HR). The probe ID used were listed below: 207113_s_at (TNF), 205225_at (ESR1), 213693_s_at (MUC1), 202724_s_at (FOXO1)

To estimate the prognostic significance of abnormal expressed TNF, ESR1, MUC1 and FOXO1, the survival time (include OS, PFS and PPS) and gene expression levels were acquired from Kaplan Meier-plotter website. The analysis results showed that higher level of TNF is related to longer OS and PPS time (based on the survival curves and logrank *P* value), and higher level of ESR1 and lower level of FOXO1 are potential protective factors for ovarian cancer patients’ survival (based on the HR and 95% confidence intervals value) (Fig. 7). These three key genes may serve as potential markers for ovarian cancer prognosis evaluation.

## Discussion

Ovarian cancer is fatal, with high rate of metastasis, drug resistance and recurrence. But the progression and treatment for it remains unsolved. Recent studies focus on its proliferation, migration, and invasion showed relevance with miRNAs [17–19], lncRNAs [20, 21], and EMT (epithelial-mesenchymal transition) [22]. And there are also studies focus on treatment for it, such as PARP inhibitors [23, 24], hyperthermic intraperitoneal chemotherapy [25] and nanotherapy, but until now, survival rate of ovarian cancer remains low. Inspiringly, more and more microarray were applied for the investigation of its progression and drug resistance [26, 27] presented us with massive information.

In our study, three datasets were used and 681 HOUP genes and 337 HEDW genes were identified. The HOUP genes were enriched in biological processes of positive regulation of biological process, positive regulation of cellular process, cellular process, response to organic substance, response to stimulus, it is reasonable. Cell component analysis for the HOUP genes showed enrichment in cytoplasmic part, cytoplasm, intracellular part, intracellular, cell. For molecular function, significant enrichment was found in protein binding, binding, enzyme binding, carbohydrate derivative binding, catalytic activity. The analysis is also reasonable because material and energy metabolism and protein binding are common activities in tumor cells including ovarian cancer. KEGG pathways analysis for the HOUP genes showed enrichment in Rap1 signaling pathway, biosynthesis of amino acids, endocrine resistance, apoptosis, pathways in cancer, immune system, innate immune system, cytokine signaling in immune system, signal transduction, signaling by receptor tyrosine kinases. It is also reasonable because immune, cytokine, biosynthesis, apoptosis in accordance with the nature of cancer. KEGG pathways analysis for the HEDW genes showed enrichment in pathways in cancer, focal adhesion, sphingolipid signaling pathway, EGFR tyrosine kinase inhibitor resistance, cellular senescence, negative regulation of the PI3K/AKT network, metabolism, metabolism of lipids, signal transduction, diseases of signal transduction. Related studies about sphingolipid [28], EGFR tyrosine kinase inhibitor [29], cellular senescence [30], PI3K/AKT network [31] and metabolism of lipids [32] in ovarian cancer were found. In summary, our biological analysis results were logical and in accordance with previous researches.

GEPIA platform were used to validate of the mRNA expression of 9 HOUP hub genes, named TNF, UBC, SRC, ESR1, CDK1, PECAM1, CXCR4, MUC1 and IKBKG, with data from TCGA and GTEx, TNF, ESR1, CDK1, CXCR4 and MUC1 were confirmed significantly higher in ovarian cancer samples than that in normal samples. To test whether DNA hypomethylation caused abnormal high expression, we explored the cBioPortal platform, and TNF, ESR1, MUC1 were confirmed with negative correlations with mRNA levels (Table 1). The mRNA expression of 9 HEDW hub genes, i.e., BDNF, CDC42, CD44, PPP2R5C, PTEN, UBB, BMP2, FOXO1, KLHL2, were also validated using GEPIA. FOXO1 was significantly lower in ovarian cancer samples than that in normal samples. Then cBioPortal platform was used again, and FOXO1 were confirmed with negative correlation with their mRNA levels (Table 1).

TNF, ESR1, MUC1 are HOUP genes, suggesting a potential role in ovarian cancer progression. According to the literature, TNF is widely studied in a variety of cancers, including ovarian cancer, it is also a major mediator of inflammation, there are also report trying to combine its effect in inflammation and carcinogenesis [33, 34], while no report concerning methylation of TNF in ovarian cancer was found. ESR1, reported in many solid malignancies [35], is frequently methylated in ovarian cancer cell lines [36], its methylation was also detected in cfDNA of high-grade serous ovarian cancer patients [37]. High expression of MUC1 is associated with epithelial ovarian cancer progression [38], hypomethylated MUC1 in poorly-differentiated ovarian cancer indicated functions in tumorigenesis, metastasis, invasion and migration [39], and a combination of MUC1 vaccination and anti-PD-L1 blockade resulted improved survival in ovarian cancer patients [40].

FOXO1, a HEDW genes, effects in many carcinomas including ovarian cancer [41], it is also related to drug resistance in ovarian cancer [42, 43], while it is not reported in ovarian cancer concerning methylation of FOXO1.

## Conclusions

Taken together, with the datasets of gene expression and methylation, our study presented an integrated bioinformatics analysis of abnormally methylated DEGs for ovarian cancer. Hub genes including TNF, ESR1, MUC1 and FOXO1 might be potential targets for diagnosis or treatment of ovarian cancer in an epigenetic approach, TNF, ESR1 and FOXO1 may serve as potential markers for ovarian cancer prognosis evaluation.

## Data Availability

The datasets analyzed during the current study are available in the GEO repository, https://www.ncbi.nlm.nih.gov/geo/query/acc.cgi?acc=GSE26712 https://www.ncbi.nlm.nih.gov/geo/query/acc.cgi?acc=GSE66957 https://www.ncbi.nlm.nih.gov/geo/query/acc.cgi?acc=GSE81224
